# Endocrine Late Effects of Targeted and Immune-Based Therapies in Pediatric Oncology

**DOI:** 10.3390/cells15080676

**Published:** 2026-04-11

**Authors:** Vittorio Ferrari, Alice Ranieri, Alessandro Ruggi, Marcello Lanari, Fraia Melchionda, Arcangelo Prete, Federico Baronio

**Affiliations:** 1 Pediatric Unit, IRCCS Azienda Ospedaliero-Universitaria di Bologna, 40138 Bologna, Italy; marcello.lanari@unibo.it (M.L.); federico.baronio@aosp.bo.it (F.B.); 2 Specialty School of Pediatrics, Alma Mater Studiorum, University of Bologna, 40126 Bologna, Italy; alice.ranieri3@studio.unibo.it; 3 Pediatric Hematology and Oncology, IRCCS Azienda Ospedaliero-Universitaria di Bologna, 40126 Bologna, Italy; alessandro.ruggi@aosp.bo.it (A.R.); fraia.melchionda@aosp.bo.it (F.M.); arcangelo.prete@unibo.it (A.P.); 4 Department of Medical and Surgical Sciences, Alma Mater Studiorum, University of Bologna, Via Massarenti 11, 40126 Bologna, Italy

**Keywords:** pediatric oncology, endocrine late effects, targeted therapies, immunotherapies, CAR T-cell therapy

## Abstract

Advances in pediatric oncology have markedly improved survival, shifting attention toward long-term treatment-related morbidity. Targeted agents and immune-based therapies are now widely used across pediatric malignancies and selected non-malignant conditions, often for prolonged periods and during critical windows of growth and development. Because many therapeutic targets regulate physiological pathways involved in growth, pubertal maturation, gonadal function, bone metabolism, and energy homeostasis, clinically relevant endocrine toxicity may emerge during treatment or become apparent only with extended follow-up. This narrative review summarizes pediatric evidence on endocrine and metabolic effects associated with major classes of targeted and immune-based therapies, including tyrosine kinase inhibitors, mTOR inhibitors, MAPK-pathway inhibitors (BRAF/MEK), TRK inhibitors, ALK inhibitors, immune checkpoint inhibitors, and immune effector therapies. Distinct patterns of endocrine vulnerability emerge across drug classes: growth impairment and bone–mineral alterations are most consistently reported with tyrosine kinase inhibitors; weight gain and metabolic changes predominate with MAPK-, TRK-, and ALK-targeted agents; immune checkpoint inhibitors are characterized by early, multi-axis immune-related endocrinopathies with a high likelihood of permanent hormone deficiency once established. In contrast, endocrine abnormalities observed after immune effector therapies largely reflect indirect effects of systemic inflammation, corticosteroid exposure, and prior hematopoietic stem cell transplantation rather than direct endocrine toxicity. Given the limited pediatric-specific data, frequent confounding by multimodal therapy, and the potential for delayed or irreversible endocrine sequelae, structured endocrine monitoring and long-term survivorship care are essential for children exposed to modern anticancer therapies.

## 1. Introduction

Over the past decades, advances in pediatric oncology have substantially improved survival, creating a growing population of childhood cancer survivors who require structured long-term follow-up. Therefore, clinical focus has progressively moved beyond acute treatment-related toxicities toward long-term morbidity, which is a major determinant of long-term health and quality of life.

Historically, endocrine sequelae in pediatric oncology have been primarily attributed to conventional cytotoxic chemotherapy and radiotherapy, with possible growth impairment, hypothalamic–pituitary dysfunction, thyroid disease, gonadal failure, and metabolic alterations [1,2,3]. Their pathophysiology, latency, and clinical management have been extensively characterized, and existing survivorship follow-up strategies are largely built around these traditional treatment exposures [4].

Over the last two decades, treatment paradigms have shifted markedly. Targeted therapies and immune-based treatments are increasingly used in both malignant and selected non-malignant pediatric conditions, often administered over prolonged periods of time or sometimes indefinitely. For example, tyrosine kinase inhibitors (TKIs) have significantly improved outcomes in diseases such as Philadelphia chromosome-positive leukemias [5,6,7], NTRK-fusion positive tumors [8], and ALK-driven malignancies, such as ALK-positive anaplastic large cell lymphoma [9]. Likewise, BRAF and MEK inhibitors [10,11,12,13] are now a key part of the management of pediatric low-grade gliomas. Many other molecularly targeted and immune-based therapies, including mechanistic target of rapamycin (mTOR) inhibitors and immune checkpoint inhibitors (ICIs), are increasingly used for pediatric patients [14,15].

These approaches often reduce exposure to conventional chemotherapy and can meaningfully improve tolerability and quality of life. Nonetheless, this does not exclude the risk for relevant endocrine side effects. Many targets inhibited by these therapies, such as breakpoint cluster region-Abelson proto-oncogene fusion protein (BCR-ABL), platelet-derived growth factor receptor (PDGFR), KIT proto-oncogene receptor tyrosine kinase (c-KIT), and components of the mTOR pathway, play a role in growth, puberty, gonadal function, bone remodeling, and metabolic homeostasis [16]. Immune checkpoint inhibition can disrupt endocrine homeostasis through immune-mediated damage to endocrine tissues, leading to clinically significant and often permanent hormone deficiencies once established [14].

**Figure 1:**
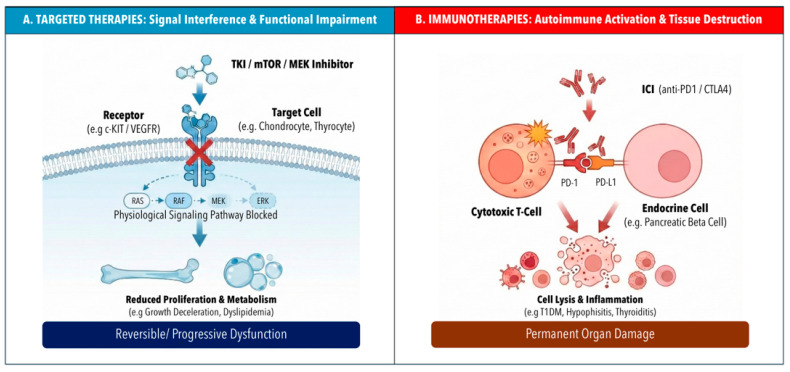
Distinct pathophysiological mechanisms of endocrine toxicity induced by targeted therapies versus immune checkpoint inhibitors. (**A**) Targeted agents, such as tyrosine kinase inhibitors (TKIs), mTOR inhibitors, and MEK inhibitors, exert endocrine effects primarily through the inhibition of physiological signaling pathways. These drugs may bind to specific receptors (e.g., c-KIT, VEGFR, PDGFR) expressed on non-malignant target cells, such as chondrocytes or thyrocytes, or may act by blocking downstream intracellular cascades (e.g., the RAS/RAF/MEK/ERK pathway). This may have multiple endocrine effects depending on the specific tissues that are targeted by the drug, such as altered bone metabolism, dyslipidemia, growth impairment, etc. These effects are usually dose-dependent and potentially reversible upon treatment discontinuation. (**B**) Immune checkpoint inhibitors (ICIs), such as anti-PD-1 or anti-CTLA-4 antibodies, induce endocrine toxicity by disrupting immune tolerance. Under physiological conditions, the interaction between immune checkpoints (e.g., PD-1 on T-cells and PD-L1 on somatic cells) acts as a “brake” to prevent autoimmunity. ICIs block this interaction, with possible endocrine toxicity through cytotoxic T-cell activation and autoimmunity (e.g., pancreatic beta-cells, thyrocytes, or pituitary cells). This may lead to permanent organ failure (e.g., type 1 diabetes mellitus or hypophysitis) requiring life-long hormone replacement.

In addition, immune effector therapies increasingly used in pediatric hematologic malignancies—including CD19-directed bispecific T-cell engagers, antibody–drug conjugates, and chimeric antigen receptor (CAR) T-cell therapies—may also contribute to endocrine morbidity, predominantly through treatment-related immune activation, cytokine-mediated stress responses, prolonged corticosteroid exposure, and hematopoietic stem cell transplantation (HSCT)-related sequelae rather than through direct endocrine tissue toxicity (Figure 1).

The pediatric population is uniquely vulnerable to these effects. Exposure frequently occurs during critical windows of growth and development, treatment duration may extend for years or even decades, and subtle endocrine alterations can translate into clinically relevant consequences only after a long latency. Pediatric studies have documented growth deceleration, alterations of the growth hormone–insulin-like growth factor 1 (GH–IGF-1) axis, thyroid dysfunction, gonadal effects, and metabolic disturbances in children treated with TKIs, with patterns that differ from those observed in adults and are strongly influenced by age at exposure, pubertal status, and treatment duration [16,17,18]. Emerging pediatric data on immune checkpoint inhibitors similarly indicates the involvement of multiple endocrine axes, with variable timing of onset and a high likelihood of irreversibility once dysfunction becomes clinically apparent [14].

Despite growing recognition of these issues, the evidence based on endocrine late effects of targeted and immune-based therapies in pediatric oncology remains limited. Available data are frequently derived from small or retrospective studies, with relatively short follow-up, making incidence estimates, risk stratification, and evidence-based surveillance strategies difficult to define. Interpretation of the available pediatric literature is further complicated by confounding from prior and concurrent exposures, particularly to radiotherapy, alkylating agents, corticosteroids, and hematopoietic stem cell transplantation (HSCT), since many children receiving targeted or immune-based agents are treated in relapsed/refractory settings or within multimodal protocols, making causal attribution and incidence estimates challenging.

In this narrative review, we summarize the available evidence on endocrine effects of targeted and immune-based therapies in pediatric oncology, organizing findings by pharmacological class and endocrine axis to provide an integrated overview, highlight clinically relevant patterns, and identify key knowledge gaps.

Table 1 provides a summary of the main targeted and immune-based therapies discussed in this review and their principal pediatric malignancies/clinical settings.

## 2. Literature Search Strategy

A literature search was conducted in PubMed/MEDLINE to identify studies reporting endocrine and/or metabolic effects associated with targeted and immune-based therapies in pediatric and adolescent populations.

Search terms combined drug-class keywords (e.g., tyrosine kinase inhibitors; TRK inhibitors; BRAF and MEK inhibitors; ALK inhibitors; mTOR inhibitors; immune checkpoint inhibitors) with endocrine and metabolic outcome terms (e.g., endocrine; thyroid; pituitary; adrenal; gonadal; growth; puberty; diabetes; glucose; lipids; bone; mineral metabolism). To capture chronic and late effects, terms related to prolonged exposure and long-term outcomes (e.g., long-term; late effects; chronic toxicity; prolonged treatment; survivorship) were included.

The search was restricted to human studies and focused primarily on studies involving pediatric and adolescent patients; when pediatric clinical data were sparse, selected preclinical or adult studies were considered only to support mechanistic interpretation, and were not used to estimate incidence. Publications from 1 January 1990 to 31 December 2025 were eligible. Original articles, clinical trials, observational studies, pooled analyses, case series, case reports, and relevant narrative reviews were considered eligible. Given the heterogeneity of study designs, patient populations, prior therapeutic exposures, and endocrine endpoints, together with the limited pediatric-specific evidence for several drug classes, a narrative synthesis was undertaken, organizing findings by pharmacologic class and endocrine axis, and emphasizing clinically relevant patterns (timing of onset, reversibility, and monitoring implications).

Reference lists of included studies and relevant reviews were manually screened to identify additional eligible publications not retrieved by electronic search.

## 3. Endocrine Effects by Pharmacological Class

### 3.1. BCR::ABL1 Tyrosine Kinase Inhibitors

*BCR*::*ABL1* TKIs represent one of the earliest and most extensively used classes of targeted therapies in pediatric oncology. Their introduction has profoundly altered the clinical course of diseases such as chronic myeloid leukemia (CML) and other Philadelphia chromosome-positive leukemias. In the case of CML, the introduction of TKIs has produced a dramatic improvement of prognosis, with a life expectancy comparable to that of the general population [19,20,21,22,23]. Also, the prognosis of Philadelphia-positive pediatric acute lymphoblastic leukemia has dramatically improved since the introduction of TKIs, with many patients being cured without the need for HSCT [6,24]

Although TKIs were designed to selectively inhibit oncogenic kinases, most agents currently used in clinical practice display a broader inhibitory profile. Drugs such as imatinib, dasatinib, nilotinib, bosutinib, and ponatinib also target kinases including c-ABL, c-KIT, PDGFR, and related signaling molecules [16]. These kinases are involved in physiological processes such as growth plate regulation, bone remodeling, gonadal development, and metabolic homeostasis, providing a biological rationale for endocrine and metabolic adverse effects observed during long-term exposure.

Pediatric cohorts indicate that the endocrine effects of TKIs differ from those observed in adults, with vulnerability influenced by age at exposure, pubertal status, and treatment intensity [16,17,18]. Impairment of linear growth represents the most consistently reported endocrine effect in pediatric patients, while additional alterations involving multiple endocrine axes have also been described [16].

#### 3.1.1. Growth Impairment and Growth Hormone–Insulin-like Growth Factor 1 Axis Dysfunction

Exposure to TKIs during childhood is frequently accompanied by clinically relevant effects on linear growth. Multiple observational studies, registry analyses, and case series have documented a significant decline in height velocity during TKI therapy, particularly with imatinib in pediatric CML, with a reduction in height standard deviation score observed in approximately 70–75% of patients, most pronounced in prepubertal children [16]. Data from the German CML-PAED registry show a significant reduction in height SDS after 12 months of imatinib therapy, with further decline at 24 months; younger age at treatment initiation, prepubertal status, and higher systemic exposure were independently associated with greater growth impairment [18]. Similar patterns have been reported in other retrospective series, confirming that growth deceleration represents an early and progressive phenomenon during TKI exposure [25,26].

The biological mechanisms underlying TKI-associated growth impairment appear to reflect a direct effect on the GH–IGF-1 axis. In a cross-sectional study specifically designed to investigate endocrine alterations in children treated with imatinib, all patients exhibited at least one abnormality of the GH–IGF-1 axis, including GH deficiency, GH insensitivity, or reduced IGF-1 and insulin-like growth factor–binding protein 3 (IGFBP-3) levels, which correlated with both treatment duration and the degree of growth impairment [17]. Notably, classical endocrine deficiencies are not uniformly present. Several studies report normal baseline thyroid, gonadal, and adrenal function, as well as preserved nutritional status, despite significant growth deceleration [17,27]. This observation suggests that TKIs may interfere directly with growth plate physiology and intracellular signaling pathways critical for chondrocyte proliferation and differentiation, rather than inducing growth failure through hormonal dysfunction [16].

The potential for growth recovery after treatment modification or discontinuation remains incompletely defined. Partial catch-up growth has been reported in some patients, particularly during puberty, although it is often unclear whether final adult height reaches the genetically predicted target [27]. A recent case report provides proof-of-concept that growth impairment may be at least partially reversible: in a child with CML treated long-term with dasatinib, the introduction of growth hormone therapy resulted in a marked increase in growth velocity and catch-up growth, without compromising disease control [28] However, evidence on the safety and efficacy of GH therapy in this population is extremely limited, and its use should not be considered routine.

#### 3.1.2. Bone Metabolism and Mineral Homeostasis

Alterations in bone metabolism and mineral homeostasis have been described in pediatric patients treated with TKIs, particularly imatinib. These effects are supported by the known involvement of several kinases inhibited by TKIs—including PDGFR, c-ABL, c-FMS, and c-KIT—in osteoblast and osteoclast function, as well as in growth plate physiology [16].

In adult populations, treatment with imatinib and other BCR–ABL inhibitors has been associated with increased trabecular bone volume, altered bone remodeling, and changes in mineral density [29,30]. Small pediatric series have reported reductions in bone mineral density in children treated with imatinib, although these findings are not consistent across studies and are often limited by small sample size and lack of appropriate correction for height SDS [31,32,33].

Disorders of calcium–phosphate metabolism represent a recurrent finding. In adult cohorts, hypophosphatemia has been reported in more than half of patients treated with imatinib, often emerging within the first months of therapy and persisting during long-term exposure [29,34,35,36,37]. Pediatric data are more limited but suggest a similar pattern. In a study including 17 children treated with imatinib, hypocalcemia was observed in 25% of patients, while vitamin D insufficiency or deficiency was documented in more than half of the cohort and was frequently associated with secondary hyperparathyroidism [38]. These abnormalities were frequently detected in the absence of clinically evident skeletal manifestations.

Several mechanisms have been proposed to explain these alterations. Experimental data suggest that imatinib interferes with osteoclast differentiation through the inhibition of colony-stimulating factor 1 receptor (c-FMS) while simultaneously affecting osteoblast activity via PDGFR and c-ABL signaling [16]. In addition, inhibition of 25-hydroxyvitamin D 1α-hydroxylase (CYP27B1) has been hypothesized as a mechanism contributing to reduced synthesis of active vitamin D, providing a potential explanation for the occurrence of vitamin D deficiency and secondary hyperparathyroidism [39].

Preclinical data from animal models further support a direct effect of TKIs on bone development. In juvenile rats, imatinib markedly impairs the growth of bone length (including vertebral height), consistent with clinical growth impairment seen in pediatric patients, whereas dasatinib and bosutinib show smaller or no effects in this model [40].

#### 3.1.3. Thyroid Dysfunction

TKIs have been associated with both hypothyroidism and hyperthyroidism, with heterogeneous clinical presentation and variable timing of onset.

A large pharmacovigilance analysis combining data from the Food and Drug Administration (FDA) Adverse Event Reporting System and published literature (not restricted to pediatric populations) identified 326 cases of thyroid dysfunction associated with BCR–ABL TKIs, most commonly hypothyroidism (74%), with less frequent cases of hyperthyroidism or a biphasic course characterized by initial thyrotoxicosis followed by hypothyroidism; onset was highly variable, occurring most often within the first nine months of treatment but ranging from a few days to several years after therapy initiation, and thyroid dysfunction was usually de novo when baseline data were available [41].

Pediatric-specific data on TKI-associated thyroid dysfunction remain limited but are consistent with observations in adult populations. In pediatric cohorts treated with imatinib, dasatinib, or nilotinib, alterations of thyroid function are often subclinical and detected through routine biochemical monitoring rather than overt symptoms [16]. In children receiving levothyroxine replacement prior to TKI initiation, an increase in TSH levels and a need for dose escalation have been reported, indicating a potential interaction between TKIs and thyroid hormone metabolism or clearance [42].

Several mechanisms have been proposed to explain TKI-related thyroid dysfunction. These include interference with thyroid angiogenesis mediated by PDGFR and vascular endothelial growth factor receptor (VEGFR) inhibition, direct toxic effects on thyroid follicular cells, immune-mediated thyroiditis, impaired iodine uptake, and increased peripheral metabolism or clearance of thyroid hormones [43].

In most reported cases, thyroid dysfunction could be managed with standard medical therapy without permanent discontinuation of the TKI, although dose adjustment or temporary interruption was sometimes required [41].

#### 3.1.4. Gonadal Axis and Reproductive Function

Data on gonadal and reproductive effects of TKIs in pediatric patients are limited compared with those on growth and bone metabolism. Nevertheless, available clinical and experimental evidence suggests potential TKI-related effects on the gonadal axis.

In pediatric and adolescent males treated with imatinib, serum testosterone, luteinizing hormone (LH), follicle-stimulating hormone (FSH), and inhibin B levels generally remain within age- and Tanner stage-adjusted reference ranges, with spontaneous pubertal progression in patients entering puberty during therapy. Despite largely reassuring endocrine profiles, concerns regarding spermatogenesis have emerged.

In a study by Chang et al., patients with chronic-phase CML treated with imatinib exhibited reduced sperm density, counts, survival rates and activity, with normal sex hormone levels [44]. Ganju et al. [45] also found imatinib therapy to be associated with a decline in sperm concentration, motility, vitality, and normal morphology, again with normal mean hormone levels.

Experimental data support a potential vulnerability of the prepubertal testis to TKI exposure. Animal studies demonstrate that early-life exposure to imatinib can interfere with gonocyte migration, spermatogonial stem cell proliferation, and Leydig cell maturation, particularly when exposure occurs before or during puberty [46,47,48]. In rat models, long-term exposure to imatinib and dasatinib has been associated with non-significant reductions in testosterone levels in postpubertal animals, while inhibin B levels remain largely preserved [49].

Based on these observations, counseling male patients and their families regarding potential reproductive risks, including the consideration of semen cryopreservation prior to initiation of long-term TKI therapy in postpubertal adolescents, might be considered.

Data on the effects of TKIs on female gonadal function in pediatric patients are limited. Most available information derives from adult cohorts or mixed-age populations. In women treated with imatinib, menstrual irregularities, reduced fertility, and adverse pregnancy outcomes have been reported, with up to 10–20% of maternal exposures during the 1st trimester ending in fetal problems or abortion [50,51]. Women should be advised to use effective contraception during TKI treatment [50,52]

Pediatric-specific endocrine data on ovarian function during TKI therapy are limited. Normal pubertal development has been reported in adolescent females treated with TKIs, although systematic hormonal assessments were not available [16]. Given the lack of longitudinal pediatric data, the long-term impact of chronic TKI exposure on ovarian reserve remains undefined.

#### 3.1.5. Metabolic Alterations

Although initially considered metabolically neutral, several TKIs—particularly second-generation BCR–ABL inhibitors—have been associated with alterations in glucose and lipid metabolism. Hyperglycemia has been reported in patients treated with imatinib, dasatinib, and nilotinib, with heterogeneous patterns across different agents. In a large retrospective study of patients with CML, hyperglycemia occurred more frequently and earlier in those treated with dasatinib or nilotinib than with imatinib, affecting 25.6%, 18.6%, and 12.4% of patients, respectively; multivariate analysis identified dasatinib and nilotinib as independent predictors of reduced hyperglycemia-free survival, supporting a drug-specific effect [53]. However, discrepant findings have also been reported, with some studies describing improvements in glycemic control or hypoglycemic effects, particularly in patients with pre-existing diabetes; these differences have been attributed to variation in patient populations, baseline metabolic status, duration of follow-up, and criteria used to define metabolic outcomes [54,55,56].

Alterations in lipid metabolism were also observed, with hypertriglyceridemia most prominent in patients treated with dasatinib (23.3%) compared with nilotinib (14.7%) and imatinib (11.2%); dasatinib emerged as the main independent risk factor, even in patients without pre-existing metabolic abnormalities [53]. Nilotinib was more strongly associated with increases in total and LDL cholesterol and has been linked to dyslipidemia and increased cardiovascular risk in multiple studies [57,58,59,60].

Several mechanistic hypotheses have been proposed to explain TKI-associated metabolic alterations. Experimental and clinical data suggest that the inhibition of c-ABL signaling may interfere with insulin receptor pathways, contributing to insulin resistance at a post-receptor level [61]. In addition, c-KIT signaling has been implicated in pancreatic β-cell survival and function, and its inhibition has been associated with reduced β-cell mass, impaired insulin secretion, and glucose intolerance in animal models [62].

### 3.2. mTOR Inhibitors

mTOR inhibitors (mostly everolimus and sirolimus) are an established therapy for some mTOR-pathway dependent tumors, with everolimus being notably used for tuberous sclerosis complex-associated subependymal giant cell astrocytomas (SEGAs) [15,63] Their mechanism of action relies on the inhibition of mechanistic target of rapamycin complex 1 (mTORC1) signaling, a central regulator of cellular growth, metabolism, and proliferation [64].

Because mTOR signaling plays a key role in physiological growth, energy balance, skeletal development, and endocrine regulation, chronic pharmacological inhibition during childhood has raised concerns regarding potential long-term endocrine consequences. Sometimes treatment with mTOR inhibitors may be continued for years, resulting in prolonged exposure during critical phases of development.

#### 3.2.1. Growth and Physical Development

Early concerns regarding growth impairment during mTOR inhibition largely originated from pediatric transplant populations, in which growth retardation was observed in children receiving sirolimus [65,66] However, in these settings, growth outcomes were often confounded by end-stage organ disease, corticosteroid exposure, and comorbid endocrine dysfunction [65,66], and subsequent studies did not confirm this observation [67].

A retrospective analysis of the ESOSIPT consortium evaluated physical development in 120 prepubertal children with tuberous sclerosis complex treated with sirolimus and followed for at least 12 months [68]. No significant differences were observed in the proportion of children with normal height, weight, or BMI before and after treatment. After one year of therapy, most patients remained within normal reference ranges for height (94.2%), weight (95.0%), and BMI (76.7%). Importantly, no correlation was identified between sirolimus blood concentrations and changes in BMI.

Data from a large, pooled analysis including more than 1500 pediatric patients treated with sirolimus for tuberous sclerosis complex or lymphangioleiomyomatosis showed no association between long-term sirolimus exposure and impaired growth, as assessed by height, weight, and BMI trajectories [69].

Although mTOR inhibition affects muscle and metabolic pathways involved in somatic growth in experimental models [70,71,72], available pediatric data do not show a clear impact on physical development.

#### 3.2.2. Metabolic and Lipid Alterations

Alterations in glucose homeostasis and lipid metabolism represent the most consistent endocrine-related effect of mTOR inhibitors in pediatric populations, with a significant increase in the risk of hyperglycemia, hypercholesterolemia, and hypertriglyceridemia when compared with controls in a systematic review and meta-analysis of Phase II–III cancer trials by Sivendran et al. [73].

For hyperglycemia, a systematic review by Arena et al. found an incidence of 16.9% in patients treated with everolimus [74]. This is hypothesized to be derived from the direct inhibition of pancreatic β cell function and insulin secretion, with a promotion of peripheral insulin resistance (partly via mTORC2 interference), leading to impaired glucose tolerance.

Clinical experience in oncology and transplantation indicates that hyperglycemia may appear early but can also persist during chronic therapy, particularly in those with preexisting metabolic risk; it usually responds to standard antidiabetic measures and/or dose adjustment, though some patients require long-term pharmacologic treatment [73,75].

In the ESOSIPT cohort, abnormal lipid profiles were observed in approximately 17% of children treated with sirolimus, including increases in LDL cholesterol, HDL cholesterol, or triglycerides, and were not associated with BMI increase, age, or sex, suggesting that mTOR inhibitor-related dyslipidemia may occur independently of changes in somatic growth or body composition [68]. These lipid changes, particularly increases in total and LDL cholesterol, were most commonly observed during the first year of treatment and tended to stabilize thereafter; triglyceride levels remained largely stable during prolonged follow-up in pediatric patients in contrast to higher overall lipid levels observed in adults [69].

Similar metabolic alterations have been observed in children exposed to mTOR inhibitors early in life. In a retrospective study of children with tuberous sclerosis complex who initiated sirolimus therapy before two years of age, hyperlipidemia, particularly hypertriglyceridemia and hypercholesterolemia, was observed in the majority of patients, reaching 100% prevalence among children aged 1–2 years [76]. Most cases were mild to moderate and did not require hospitalization. Dietary factors appeared to modulate lipid levels, as children receiving ketogenic diets exhibited the highest lipid values, which partially improved after dietary adjustment.

Across studies, mTOR inhibitor-induced hyperlipidemia appears to be dose-dependent and potentially reversible, although long-term cardiovascular implications in pediatric patients remain poorly defined [69,76].

#### 3.2.3. Gonadal Function and Reproductive Effects

Data on gonadal function during mTOR inhibition in pediatric patients are limited and derive largely from transplant populations and mixed-age cohorts. Across pediatric renal transplant studies, everolimus exposure was not associated with alterations in sex hormone levels, whereas sirolimus was linked to a dose-dependent reduction in testosterone levels in some adolescent males, accompanied by compensatory increases in luteinizing hormone (LH) [77,78,79,80,81]. Experimental models suggest functional reversibility of spermatogenesis after drug discontinuation [82]. Studies by Kranz et al. [67] and Förster et al. [83] did not find any long-term negative impact of mTOR inhibitors on pubertal development in renal transplant recipients.

Adult data from pooled analyses indicate a substantial burden of menstrual irregularities in women treated with sirolimus, with disturbances observed in nearly half of adult female patients [69]. Whether similar ovarian toxicity occurs in adolescent girls treated during puberty remains unknown.

### 3.3. MAPK-Targeted Therapy: BRAF and MEK Inhibitors

BRAF and MEK inhibitors have assumed a central role in the contemporary treatment landscape of pediatric low-grade glioma, in which MAPK-pathway activation represents the dominant biological driver and has directly informed molecularly guided therapeutic strategies. These agents are also increasingly used in other pediatric MAPK-driven neoplasms, including selected high-grade gliomas and Langerhans cell histiocytosis (LCH).

LCH is itself a histiocytic neoplasm with frequent BRAF V600E alterations and is increasingly managed with targeted inhibitors in relapsed/refractory and selected frontline settings.

Given the central role of MAPK signaling in cellular proliferation, differentiation, energy balance, and neuroendocrine regulation, prolonged pharmacological inhibition raises concerns regarding potential endocrine and metabolic effects [84].

#### 3.3.1. Sodium Homeostasis

Disturbances of sodium balance have emerged as a relevant endocrine-related adverse effect in pediatric patients treated with MEK inhibitors. In a retrospective single-center cohort of pediatric patients treated with BRAF and/or MEK inhibitors, hyponatremia was observed in approximately 16% of cases and occurred more frequently in patients receiving trametinib [84]. Importantly, during treatment, children with pre-existing central diabetes insipidus experienced more pronounced reductions in serum sodium, identifying this subgroup as particularly vulnerable to treatment-associated hyponatremia [84]. This issue is especially relevant in patients with optic-hypothalamic low-grade glioma and in LCH with hypothalamic–pituitary involvement, in whom endocrine abnormalities during BRAF/MEK inhibition may reflect pre-existing hypothalamic–pituitary damage, treatment-related toxicity, or an interaction between the two.

Egan et al. have also reported on two pediatric cases of severe hyponatremia occurring after the initiation of trametinib in children with optic-hypothalamic low-grade gliomas and pre-existing central diabetes insipidus [85].

Although the underlying mechanism remains incompletely understood, MEK inhibition has been hypothesized to potentiate antidiuretic hormone action through the modulation of aquaporin trafficking [85]. Experimental studies have shown that MAPK signaling influences aquaporin insertion in renal medullary cells [86,87], and that desmopressin reduces extracellular signal-regulated kinases 1 and 2 (ERK1/2) phosphorylation downstream of MEK in collecting duct cells [88]. These findings raise the possibility that MEK inhibition may enhance the antidiuretic effect of desmopressin, supporting the need for close sodium monitoring and dose adjustment in patients with diabetes insipidus or optic-hypothalamic low-grade glioma that receive MEK inhibitors.

#### 3.3.2. Glucose Metabolism

Alterations in glucose homeostasis have been reported in pediatric patients treated with BRAF and MEK inhibitors. In a retrospective cohort, abnormalities of glucose regulation, including insulin resistance or impaired glucose tolerance, were identified in six patients, with four diagnoses occurring after the initiation of dabrafenib therapy [84]. All affected patients had underlying hypothalamic involvement, which is independently associated with obesity and insulin resistance. While this limits the ability to attribute glucose metabolism abnormalities solely to treatment, the temporal association with drug initiation indicates that treatment-related effects on glucose homeostasis cannot be excluded and may coexist with tumor-related hypothalamic dysfunction [89,90].

Nevertheless, adult data indicate that hyperglycemia is a recognized adverse effect of dabrafenib, with reported rates of up to 46% in thyroid cancer [91].

BRAF inhibition may affect glucose uptake in BRAFV600E-mutated melanoma cells, with effects on glucose transporters glucose transporter type 1 (GLUT1) and glucose transporter type 3 (GLUT3) [92,93]. In addition, disruption of MAPK/ERK signaling may interfere with melanocortin-4 receptor pathways involved in energy homeostasis and obesity [94]. Whether these mechanisms contribute to glucose dysregulation in pediatric patients without pre-existing hypothalamic injury remains unclear.

Further support for a potential metabolic signal derives from a phase 2 trial in patients with newly diagnosed BRAF V600E-mutated papillary craniopharyngioma, in which one case of grade 4 hyperglycemia was reported among the treatment-related adverse events [95].

#### 3.3.3. Body Weight Regulation

Changes in body weight represent the most frequently reported metabolic effect of MAPK-targeted therapy in pediatric populations. In a randomized, open-label phase 2 trial comparing dabrafenib plus trametinib with standard chemotherapy in children with BRAF V600-mutated low-grade glioma, an increase of at least two BMI-for-age percentile categories was observed in 44% of patients receiving targeted therapy. Notably, weight gain occurred at similar rates in patients with hypothalamic or optic pathway involvement and in those with tumors at other sites, supporting a treatment-related effect rather than a consequence of tumor location [11].

Consistent findings were reported in a large retrospective cohort of 67 pediatric patients treated with MEK inhibitors for at least six months, predominantly trametinib and selumetinib. Overall, 57% of patients experienced a significant increase in weight-for-age percentile, with a median time to maximal weight change of approximately nine months. Weight trajectories were influenced by baseline nutritional status, with underweight patients frequently normalizing BMI during treatment and obese patients showing relative stabilization or reduction in weight percentile [96]. Earlier pediatric trials of selumetinib similarly reported predominantly low-grade weight gain that did not require dose modification [13,97,98].

Taken together, these findings suggest that MEK inhibition may exert predominantly anabolic effects rather than promoting isolated adipose tissue accumulation. One proposed mechanism involves the suppression of interleukin-6 (IL-6) signaling, a pro-inflammatory pathway implicated in muscle catabolism; reductions in IL-6 levels have been associated with weight gain and increased muscle mass in other clinical contexts [96]. Consistent with this hypothesis, an adult clinical study demonstrated increased skeletal muscle mass in patients treated with selumetinib compared with standard therapy [99].

Interestingly, emerging data suggest that tovorafenib may induce a reversible growth arrest in pediatric and adolescent patients. This effect occurs without advancement of bone age or premature epiphyseal closure and has not been reported with MEK inhibitors; whether this represents a class effect remains unclear [100,101]. Notably, normal growth velocity appears to resume after treatment discontinuation [100].

### 3.4. TRK Inhibitors

TRK inhibitors are a class of targeted therapies designed for tumors harboring gene fusions involving the neurotrophic tyrosine receptor kinase genes Neurotrophic Tyrosine Receptor Kinase 1–3 (NTRK1, NTRK2, NTRK3). First-generation TRK inhibitors, such as larotrectinib and entrectinib, are highly effective in tumors with NTRK gene fusions, regardless of tumor histology [102]. In pediatric oncology, evidence mainly comes from basket trials enrolling patients based on NTRK fusions rather than tumor type, including very young children and infants.

#### 3.4.1. Body Weight Regulation

Weight gain is a common adverse effect of TRK inhibitors and is consistent with the physiological role of TRK signaling in hunger regulation. In adult populations, treatment-related weight gain has been reported in approximately 19% of patients treated with entrectinib and appears less frequent in those receiving larotrectinib [102].

In pediatric patients, available data indicate a higher frequency of weight gain. In the pediatric cohort of the STARTRK-NG study, weight gain was the most frequently reported treatment-related adverse event with entrectinib (48.8%) and the leading cause of dose reduction [103]. The integrated data of STARTRK-NG, TAPISTRY, and STARTRK-2 trials show similar results, with weight gain being the most frequently reported adverse effect (35.2%) [104].

For larotrectinib, early-phase pediatric safety studies documented that clinically significant weight gain can occur, although it was initially uncommon; in a phase 1/2 multicenter study of larotrectinib, a single case of grade 3 weight gain (4%) was reported during dose escalation [105]. The product monograph of larotrectinib, however, reports an incidence of weight gain in pediatric patients of 23% vs. 12% in adults [106].

The biological basis of TRK inhibitor-associated weight gain is supported by preclinical and human genetic evidence implicating Tropomyosin Receptor Kinase B (TRKB) signaling in appetite regulation. Impaired TRKB activity has been shown to induce hyperphagia, obesity, and hyperdipsia in murine models, and de novo mutations affecting TRKB in humans have been associated with severe obesity and neurodevelopmental delay [107,108,109].

#### 3.4.2. Bone Homeostasis

Emerging pediatric data suggest a potential impact of TRK inhibition on bone homeostasis. In an integrated pediatric safety analysis of entrectinib, fractures were reported in 29.7% of treated patients [104]. Notably, more than half of the patients who experienced fractures showed a clinically relevant increase in BMI, progressing to overweight or obese categories compared with the baseline, raising the possibility that altered mechanical loading and metabolic status may contribute to fracture risk.

Preclinical studies indicate that TRK receptors are widely expressed in skeletal tissues and play a role in chondrogenesis, osteoblastogenesis, osteoclastogenesis, and the regulation of bone formation and remodeling [110,111,112]. However, fracture risk is likely multifactorial and may be influenced by overweight status as well as underlying disease-related factors [113,114,115].

### 3.5. ALK Inhibitors

ALK inhibitors have been introduced into pediatric oncology for the treatment of ALK-driven malignancies, including anaplastic large cell lymphoma, neuroblastoma, and selected solid tumors. Pediatric safety data have historically focused on hepatic, gastrointestinal, neurological, and cardiovascular toxicity, while endocrine outcomes have been less systematically evaluated [16,116]. Nevertheless, emerging pediatric evidence suggests that ALK inhibition, particularly with newer-generation agents, may be associated with clinically relevant metabolic and endocrine-related effects.

The most prominent endocrine-related effects reported to date are metabolic, particularly weight gain and dyslipidemia, and have been most clearly described with lorlatinib. In a multicenter phase 1 study of pediatric and adult patients with relapsed or refractory ALK-driven neuroblastoma, hypertriglyceridemia, hypercholesterolemia, and weight gain were the most frequently reported adverse effects, occurring in approximately 90%, 79%, and 87% of patients, respectively. Although one pediatric patient with a germline ALK mutation discontinued treatment because of excessive weight gain, these metabolic toxicities were generally manageable with dietary counseling, supportive care, and lipid-lowering therapy [117].

Before the introduction of newer-generation ALK inhibitors such as lorlatinib, early pediatric experience with ALK inhibition—mainly derived from studies of crizotinib—documented only mild metabolic and electrolyte abnormalities. In a Children’s Oncology Group (COG) ) Phase I trial for refractory solid or central nervous system tumors, or anaplastic large cell lymphoma, hyperglycemia was reported in up to 14% of patients, while hypocalcemia and hypophosphatemia were observed in up to 19% and 14% of patients, respectively [118]. These alterations were generally transient but suggest early perturbations of metabolic and mineral homeostasis during ALK inhibition.

### 3.6. Immune Checkpoint Inhibitors

ICIs have been increasingly introduced into pediatric oncology over the past decade, particularly for relapsed or refractory Hodgkin’s lymphoma [119]. Unlike targeted therapies that directly interfere with oncogenic signaling pathways, ICIs act by modulating immune tolerance and enhancing antitumor immune responses. Given their mechanism of action, they can cause so-called immune-related adverse events (irAEs). Among these, endocrine complications are one of the most commonly observed: they have a median latency of approximately 9 weeks, but they might have a late presentation (>1 year) [120,121].

Pediatric data remain limited but suggest involvement of similar endocrine axes, with substantial variability in reported incidence, timing of onset, and clinical severity; available evidence is largely derived from Phase I–II trials and retrospective multicenter analyses [120,121,122,123,124]. The most common endocrinopathies associated with ICI use are thyroid dysfunction, diabetes mellitus, adrenal insufficiency, and hypophysitis [120,121].

#### 3.6.1. Thyroid Dysfunction

Thyroid dysfunction is the most frequently reported endocrine complication in pediatric patients treated with ICIs. Across pediatric studies, hypothyroidism has been reported in approximately 1–20% of patients, while hyperthyroidism occurs in 1–15% [119,125,126,127,128].

Thyroid abnormalities typically emerge within weeks to months after the initiation of therapy, although delayed onset has also been described [14]. Consistent with adult cohorts, inhibitors of the Programmed Death-1/Programmed Death-Ligand 1 (PD-1/PD-L1) pathway are associated with a higher incidence of thyroid dysfunction compared with anti-Cytotoxic T-Lymphocyte-Associated Protein 4 (CTLA-4) agents, while combination regimens confer the highest risk [119,129,130,131,132,133]. The most common clinical pattern is immune-mediated destructive thyroiditis, often presenting with transient thyrotoxicosis followed by permanent hypothyroidism [134].

In a retrospective cohort of pediatric brain tumor patients treated with ICIs, hypothyroidism was the only endocrine disorder identified after treatment initiation, occurring in 9.1% of patients, a frequency comparable to that reported in adult patients treated with ICI monotherapy. However, this finding should be interpreted cautiously, as pediatric brain tumor survivors are already at intrinsically increased baseline risk of thyroid dysfunction due to prior cranial or craniospinal irradiation, which complicates the attribution of causality [135].

#### 3.6.2. Hypophysitis and Pituitary Dysfunction

Pituitary dysfunction and hypophysitis are reported less frequently in pediatric patients than in adults treated with ICIs. While adult incidence rates range from approximately 3% to 10% with anti-CTLA-4 agents and increase with combination therapy, pediatric cohorts suggest that pituitary involvement is uncommon, with estimated incidences around 1–3% [121,136,137].

When present, pituitary involvement in children most commonly manifests as isolated ACTH deficiency, followed by TSH and gonadotropin deficiencies, whereas panhypopituitarism is rare [121]. Unlike adult patients, in whom hypophysitis typically occurs early during therapy, pediatric data do not allow for a reliable definition of a temporal risk window, and delayed presentations have been reported.

Available evidence indicates that pituitary hormone deficiencies are usually persistent and require long-term hormone replacement, although pituitary involvement itself does not generally necessitate the permanent discontinuation of ICI therapy once adequate endocrine control is achieved [121].

#### 3.6.3. Primary Adrenal Insufficiency

Primary adrenal insufficiency represents a very rare but potentially life-threatening endocrine complication in pediatric patients treated with ICIs. Only isolated pediatric cases have been reported, precluding reliable estimates of incidence, risk factors, or typical clinical course [121,138].

Available data suggest that adrenal insufficiency may occur relatively early during treatment, with a median onset of approximately 10 weeks after therapy initiation, potentially earlier in children than in adults [139,140]. In adult series, adrenal insufficiency more commonly occurs secondary to pituitary dysfunction, while primary adrenal involvement remains exceptional [135].

#### 3.6.4. Diabetes and Severe Hyperglycemia

Diabetes mellitus induced by immune checkpoint inhibition is a rare but severe endocrine complication. In adult populations, the reported incidence of immune checkpoint inhibitor-related diabetes mellitus ranges from 0.45% to 2%; some pediatric series report cases of diabetes, but the exact frequency is not yet known. In pediatric cohorts, hyperglycemia has been reported in 2–12% of patients, while overt diabetes mellitus occurs in approximately 1–9% [125,141,142,143]. Most cases are associated with PD-1 or PD-L1 inhibitors and typically present within the first 5–6 months of treatment. Clinical onset may be abrupt, and diabetic ketoacidosis has been reported as the initial manifestation in pediatric patients [125,135].

Importantly, this form of diabetes differs from classical childhood-onset autoimmune diabetes. C-peptide levels may be low, normal, or undetectable, and up to half of affected patients lack islet-specific autoantibodies [121,135]. Fulminant presentations appear more frequent in patients with undetectable C-peptide and positive autoantibodies [144]. These features support the recommendation that the evaluation of hyperglycemia in children treated with ICIs should include C-peptide measurement, as HbA1c levels may not rise sufficiently early in cases with rapid β-cell destruction [145].

Rarely, severe hyperglycemia driven predominantly by extreme insulin resistance has been reported in the context of combination regimens including immune checkpoint inhibitors. A single adult case treated with nivolumab in combination with brentuximab-vedotin developed profound insulin resistance associated with massive cytokine release and hemophagocytic lymphohistiocytosis, suggesting a hyperinflammatory mechanism rather than classical immune-mediated β-cell destruction [146].

### 3.7. CAR T-Cells and Other T-Cell Engaging Therapies

Immunotherapies have become a cornerstone of modern pediatric oncohematology practice, with transforming results for children and adolescents with hematologic malignancies, with recent studies also successfully applying this approach to neuroblastoma and other solid tumors [147]. Among these, immune effector therapies, such as CAR T-cell therapies, bispecific T-cell engagers (BiTEs) such as blinatumomab, and antibody–drug conjugates (ADCs) such as inotuzumab ozogamicin, are now part of the management of B-cell acute lymphoblastic leukemia [148,149,150]. The characterization of their late effects, including endocrine sequelae, is now part of survivorship care.

Unlike ICIs, these agents are not primarily associated with autoimmune endocrine gland destruction, and, to date, few durable toxicities have been directly attributable to CAR T-cell therapy itself, with the principal late effects consistently identified being hypogammaglobulinemia, prolonged cytopenias, and infectious complications [151]. However, the current consensus recommends a structured long-term surveillance of endocrine, reproductive, and bone health in CAR T-cell recipients, as these late effects may be multifactorial in origin [152].

Endocrine abnormalities observed in patients treated with immune effector therapies are largely mediated by indirect mechanisms, including systemic inflammation, cytokine release syndrome (CRS), immune effector cell-associated neurotoxicity syndrome (ICANS), repeated or prolonged high-dose corticosteroid exposure, hypothalamic–pituitary axis stress, and frequent consolidation with hematopoietic stem cell transplantation (HSCT).

CAR T-cell therapy represents the immune effector modality with the greatest apparent endocrine burden in survivorship cohorts, largely reflecting treatment intensity and prior exposures rather than a CAR T-specific mechanism. In a study by Yates et al. [153] evaluating late effects in children and young adults with B-cell acute lymphoblastic leukemia treated with CAR T-cells and/or HSCT, 56% of patients developed at least one endocrinopathy, and commonly prescribed long-term medications included vitamin D supplementation, levothyroxine, metformin, and testosterone replacement.

Overall, while immune effector therapies are not currently associated with a distinctive pattern of primary endocrine autoimmunity, they substantially contribute to the global endocrine risk profile of pediatric cancer survivors through systemic inflammation, corticosteroid exposure, and interaction with established endocrine risk factors. Endocrine surveillance in this population should therefore be exposure-driven and risk-stratified, integrating immune effector therapy within the broader context of cumulative treatment burden and long-term survivorship planning.

## 4. Surveillance & Management Considerations

Beyond the descriptive heterogeneity across drug classes, the evidence reviewed suggests that endocrine surveillance in children receiving targeted or immune-based therapies should be tailored to the expected timing and clinical phenotype of toxicity.

In children receiving TKIs, growth deceleration appears to be an early but progressive event, often becoming evident within the first year of treatment and worsening with prolonged exposure, particularly in prepubertal patients; similarly, disorders of calcium-phosphate metabolism may emerge within the first months of therapy, and thyroid dysfunction is often detected during the first 9 months, although later onset has also been reported. In this setting, endocrine abnormalities may remain clinically subtle for a prolonged period, becoming apparent only through declining height velocity, attenuation of pubertal progression, persistent fatigue, or nonspecific musculoskeletal complaints. Longitudinal surveillance should therefore include regular auxological assessment, pubertal evaluation, thyroid function testing, and periodic monitoring of calcium-phosphate balance and vitamin D status, particularly during the first year and during prolonged exposure.

In contrast, endocrine toxicity associated with ICIs tends to follow a more acute timeline and may become clinically overt within weeks to months of treatment initiation. Adrenal insufficiency may occur relatively early, with a median onset of approximately 10 weeks, whereas ICI-related diabetes most often presents within the first 5–6 months and may have an abrupt onset, including diabetic ketoacidosis. Thyroid dysfunction may also present early, sometimes through a biphasic course from thyrotoxicosis to hypothyroidism. In this setting, clinicians should maintain a high index of suspicion in the presence of fatigue, nausea, weight loss, headache, polyuria, polydipsia, or unexplained electrolyte or glycemic abnormalities, as these may represent the earliest manifestations of clinically relevant endocrine injury. These observations support closer clinical and biochemical monitoring early during treatment and prompt endocrine assessment in the presence of compatible symptoms or otherwise unexplained metabolic abnormalities. Given the substantial likelihood that these toxicities may be irreversible once clinically manifest, endocrine abnormalities occurring during ICI therapy should not be managed as transient laboratory events but as conditions potentially requiring long-term follow-up and hormone replacement.

For other targeted agents, the available pediatric evidence remains more limited. With mTOR inhibitors, the most consistent signal concerns metabolic toxicity, particularly early hyperglycemia and dyslipidemia, which appears most evident during the first year of treatment and may be accentuated in very young children or in the presence of dietary and baseline metabolic risk factors. In contrast, BRAF/MEK inhibitors are more often associated with disturbances in sodium and glucose homeostasis in selected high-risk settings, particularly in patients with hypothalamic involvement or pre-existing diabetes insipidus, while body weight increase emerges as the most recurrent metabolic effect, with maximal change often reached after several months of exposure. TRK inhibitors appear to be characterized mainly by treatment-related weight gain, likely reflecting altered appetite regulation, with an additional signal for fracture risk that may justify attention to bone health during prolonged therapy. For ALK inhibitors, the clearest pediatric signal is represented by lorlatinib-associated weight gain and dyslipidemia, whereas earlier agents such as crizotinib have been mainly linked to milder and more transient metabolic or mineral abnormalities. These observations support a drug-specific follow-up strategy, prioritizing lipid and glucose monitoring during mTOR inhibition, sodium and metabolic surveillance in children receiving BRAF/MEK inhibitors—especially when hypothalamic-pituitary vulnerability is present—attention to rapid weight change and skeletal events during TRK inhibition, and close metabolic monitoring during lorlatinib exposure.

The current pediatric evidence does not support a uniform surveillance algorithm across all targeted and immune-based therapies, nor does it yet justify fully standardized recommendations for each drug class. Nevertheless, the findings available to date support a pragmatic follow-up strategy based on the patterns of toxicity already identified while recognizing that the full spectrum and timing of endocrine sequelae remain only partially characterized. Surveillance should therefore be guided by age at exposure, pubertal stage, treatment duration, and the drug-specific signals emerging from the current literature, while maintaining awareness that additional manifestations may become clinically relevant as experience with these agents expands.

## 5. Limitations and Knowledge Gaps

The evidence on endocrine late effects of targeted and immune-based therapies in pediatric oncology remains limited. Most data derive from retrospective studies, small case series, or post hoc safety evaluations of oncological trials not designed to assess endocrine outcomes. As a result, these adverse events are often incompletely reported and not systematically assessed during longitudinal follow-up.

For tyrosine kinase inhibitors, growth and metabolic effects have been relatively well-characterized in CML cohorts, whereas data remain limited for other pediatric indications and for newer agents [17,18,53]. For mTOR inhibitors, most pediatric evidence derives from non-oncological populations or pooled analyses including heterogeneous underlying conditions [64,69]. Finally, pediatric data on ICIs largely originate from early-phase trials with small sample sizes and short follow-up, precluding strong estimates of incidence, risk factors, and long-term outcomes [14].

Across drug classes, endocrine endpoints are rarely predefined, and baseline endocrine status is often insufficiently documented. This makes it difficult to determine whether endocrine abnormalities observed during treatment are related to the therapy itself, to pre-existing conditions, to the underlying disease, or to prior treatments such as chemotherapy, radiotherapy, or hematopoietic stem cell transplantation. In addition, key modifiers of endocrine vulnerability—including age at exposure, pubertal status, cumulative dose, and duration of therapy—are frequently reported with limited detail, despite their biological relevance.

Another major gap concerns long-term and post-treatment outcomes. Many pediatric patients remain on continuous targeted therapy for years, yet data on endocrine recovery after treatment modification or discontinuation are limited or absent.

Finally, our understanding of the endocrine toxicity of targeted therapies and immunotherapies is still incomplete, and future studies might be able to explain the underlying mechanisms.

**Figure 2:**
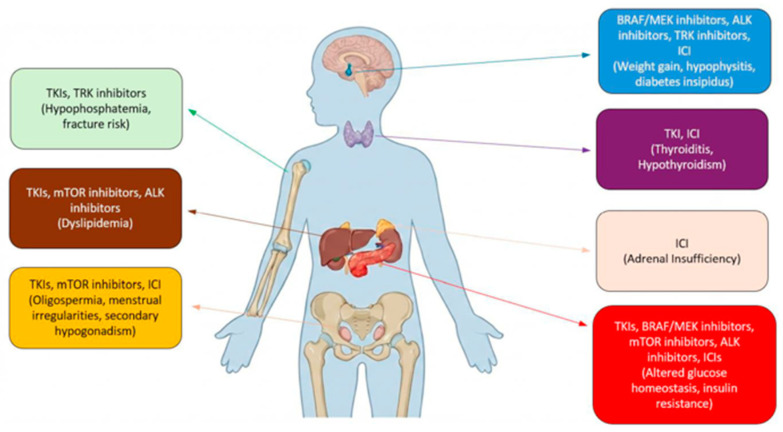
Anatomical distribution of endocrine and metabolic adverse effects associated with targeted and immune-based therapies in pediatric oncology. For each endocrine organ, a corresponding box indicates the pharmacological classes involved in the toxicity and the respective adverse effects reported in the literature.

Table 2 and Figure 2 provide an overview of the endocrine alterations associated with the targeted and immunotherapies discussed in this review.

## 6. Conclusions

Targeted and immune-based therapies have profoundly modified the treatment of pediatric cancers, contributing to longer survival and, in some cases, reducing exposure to conventional chemotherapy. As survival improves, attention to late effects becomes increasingly relevant, as was the case for endocrine complications historically associated with conventional chemotherapy and radiotherapy, which showed well-defined patterns and long-term clinical consequences.

Evidence reviewed in this work shows that newer anticancer agents are also associated with clinically relevant endocrine alterations in children, affecting growth, metabolism, pubertal development, gonadal function, and multiple hormonal axes. Although pediatric data remain limited and heterogeneous, similar endocrine effects have been reported across different clinical settings, supporting their relevance.

The observation of these endocrine alterations, even in the absence of comprehensive long-term data, already warrants careful attention. Systematic endocrine monitoring during treatment and survivorship is therefore justified based on evidence that is already emerging from pediatric studies.

Further pediatric-focused studies are needed to clarify the timing, course, and long-term consequences of endocrine adverse effects associated with modern anticancer therapies. At the same time, existing observations already support proactive endocrine surveillance in children exposed to these agents.

## Figures and Tables

**Table 1:** Main targeted and immune-based therapies discussed in the review and their principal pediatric malignancies/clinical settings.

Drug Class	Main Drugs Discussed	Main Pediatric Malignancies/Clinical Settings
**BCR::ABL1 tyrosine kinase inhibitors (TKIs)**	Imatinib, dasatinib, nilotinib, bosutinib, ponatinib	Philadelphia chromosome-positive chronic myeloid leukemia (CML) and acute lymphoblastic leukemia (Ph + ALL)
**mTOR inhibitors**	Everolimus, sirolimus	Subependymal giant cell astrocytoma (SEGA) in tuberous sclerosis complex; selected other pediatric settings
**BRAF and MEK inhibitors**	Dabrafenib, trametinib, selumetinib, tovorafenib	Pediatric low-grade gliomas; selected high-grade malignancies; Langerhans cell histiocytosis
**TRK inhibitors**	Larotrectinib, entrectinib	NTRK fusion-positive solid or central nervous system tumors
**ALK inhibitors**	Crizotinib, ceritinib, alectinib, lorlatinib	ALK-positive anaplastic large cell lymphoma; ALK-positive inflammatory myofibroblastic tumor; ALK-driven neuroblastoma (particularly relapsed/refractory disease, mainly with lorlatinib)
**Immune checkpoint inhibitors (ICIs)**	Nivolumab, pembrolizumab, ipilimumab	Selected biomarker-defined pediatric malignancies, particularly hypermutated or mismatch repair-deficient tumors (e.g., CMMRD-associated cancers); other relapsed/refractory settings under investigation
**Immune effector therapies**	Blinatumomab; CD19-directed CAR T-cell therapies	B-cell precursor acute lymphoblastic leukemia (B-ALL), particularly relapsed/refractory, and consolidation settings

**Table 2:** Overview of endocrine and metabolic alterations associated with targeted and immune-based therapies in pediatric oncology. The table summarizes the spectrum of endocrine and metabolic adverse effects reported in pediatric patients, categorized by pharmacological class and affected physiological axis. Specific clinical manifestations reported in the literature are listed for each category. Abbreviations: ALK, anaplastic lymphoma kinase; BRAF, B-Raf proto-oncogene, serine/threonine kinase; MEK, Mitogen-activated protein kinase; ICIs, immune checkpoint inhibitors; mTOR, mechanistic target of rapamycin; TKIs, tyrosine kinase inhibitors; TRK, tropomyosin receptor kinase. Symbol: (–) indicates no consistently reported data or insufficient evidence in pediatric populations; (*) indicates evidence derived mainly from adult or mixed-age cohorts, with limited or no confirmation in pediatric cohorts.

Drug Class	Linear Growth	Bone Health	Thyroid	Gonadal Axis	Pituitary	Glucose Metabolism	Weight Regulation	Electrolytes	Lipid Metabolism
**Tyrosine Kinase Inhibitors (TKIs)**	Deceleration Decline in height SDS	Altered bone architecture Hypocalcemia Hypovitaminosis D	Hypothyroidism Hyperthyroidism	Oligospermia * Menstrual irregularities * Subfertility *	–	Hyperglycemia; Hypoglycemia in previously diagnosed diabetes	–	Hypocalcemia Hypophosphatemia	Dyslipidemia
**mTOR Inhibitors**	–	–	–	Menstrual irregularities * Reduction in testosterone levels	–	Hyperglycemia	–	Hypophosphatemia	Hyperlipidemia
**BRAF & MEK Inhibitors**	–	–	–	–	–	Hyperglycemia Insulin resistance	Weight gain	Hyponatremia	–
**TRK Inhibitors**	–	Fracture risk	–	–	–	–	Weight gain	–	–
**ALK Inhibitors**	–	–	–	–	–	Hyperglycemia	Weight gain	Hypocalcemia Hypophosphatemia	Hyperlipidemia
**Immune Checkpoint Inhibitors (ICIs)**	–	–	Thyroid dysfunction	–	Hypophysitis	Hyperglycemia Diabetes Mellitus	–	–	–

## Data Availability

No new data were created in this study. Data sharing is not applicable to this article.
